# Galectin-3 Reflects Mitral Annular Plane Systolic Excursion Being Assessed by Cardiovascular Magnetic Resonance Imaging 

**DOI:** 10.1155/2016/7402784

**Published:** 2016-12-01

**Authors:** Seung-Hyun Kim, Michael Behnes, Michele Natale, Julia Hoffmann, Nadine Reckord, Ursula Hoffmann, Johannes Budjan, Thomas Henzler, Theano Papavassiliu, Martin Borggrefe, Thomas Bertsch, Ibrahim Akin

**Affiliations:** ^1^First Department of Medicine, University Medical Center Mannheim (UMM), Faculty of Medicine Mannheim, Heidelberg University, Mannheim, Germany; ^2^Institute of Clinical Radiology and Nuclear Medicine, University Medical Center Mannheim (UMM), Faculty of Medicine Mannheim, Heidelberg University, Mannheim, Germany; ^3^Institute of Clinical Chemistry, Laboratory Medicine and Transfusion Medicine, General Hospital Nuremberg, Paracelsus Medical University, Nuremberg, Germany

## Abstract

*Background*. This study investigates whether serum levels of galectin-3 may reflect impaired mitral annular plane systolic excursion (MAPSE) in patients undergoing cardiac magnetic resonance imaging (cMRI).* Methods*. Patients undergoing cMRI during routine clinical care were included prospectively within an all-comers design. Blood samples for biomarker measurements were collected within 24 hours following cMRI. Statistical analyses were performed in all patients and in three subgroups according to MAPSE (MAPSE I: ≥11 mm, MAPSE II: ≥8 mm–<11 mm, and MAPSE III: <8 mm). Patients with right ventricular dysfunction (<50%) were excluded.* Results*. 84 patients were included in the study. Median LVEF was 59% (IQR 51–64%). Galectin-3 correlated significantly with NT-proBNP (*r* = 0.42, *p* = 0.0001). Galectin-3 increased significantly according to the different stages of impaired MAPSE (*p* = 0.006) and was able to discriminate both patients with impaired MAPSE <11 mm (area under the curve (AUC) = 0.645, *p* = 0.024) and <8 mm (AUC = 0.733, *p* = 0.003). Combining galectin-3 with NT-proBNP improved discrimination of MAPSE <8 mm (AUC 0.803, *p* = 0.0001). In multivariable logistic regression models galectin-3 was still associated with impaired MAPSE (MAPSE < 11 mm: odds ratio (OR) = 3.53, *p* = 0.018; MAPSE < 8 mm: OR = 3.18, *p* = 0.06).* Conclusions*. Galectin-3 reflects MAPSE being assessed by cardiac MRI.

## 1. Introduction

Cardiac magnetic resonance imaging (cMRI) has emerged to a standard imaging modality for the diagnosis of heart failure related to coronary artery disease, cardiomyopathies, or myocarditis as well as for cardiac tissue characterization [1–3]. cMRI allows accurate assessment of cardiac function, perfusion, and vitality under resting and stress-induced circumstances due to its higher three-dimensional resolution [4]. Next to the assessment of left ventricular ejection fraction (LVEF), the so-called mitral annular plane systolic excursion (MAPSE) represents a central functional cMRI parameter reflecting left ventricular function (LVF) [5]. A reduction of MAPSE indicates impaired longitudinal LVF, whereas LVEF reflects both longitudinal and circumferential LV contractility [6]. In addition, it was shown that reduced MAPSE was associated with a poor prognosis in heart failure patients, as well as in those with atrial fibrillation and after myocardial infarction [7].

Cardiac biomarkers, such as natriuretic peptides, have been focused increasingly for the assessment of early diagnosis and short- and long-term prognosis of heart failure patients [8]. The amino terminal pro-brain natriuretic peptide (NT-proBNP) is released within the heart chambers as a consequence of myocardial stretch and volume overload, allowing to detect early and chronic stages of heart failure [9]. One study was able to demonstrate a direct correlation of NT-proBNP with MAPSE being assessed by transthoracic echocardiography [10].

Galectin-3 is a soluble ß-galactoside-binding lectin being released by activated macrophages [11]. It was demonstrated in animal models that galectin-3 contributes to the development and progression of heart failure through cardiac fibrosis and adverse structural remodeling [12]. Recent studies indicate that galectin-3 may reflect the presence of echocardiographically assessed heart failure with preserved ejection fraction (HFpEF) better than NT-proBNP due to a higher degree of cardiac fibrosis as a consequence of a chronically increased afterload during arterial hypertension [13–15]. Also galectin-3 was shown to correlate with LVEF in cMRI in patients after myocardial infarction [16] and to serve as a strong prognostic biomarker in chronic stages of heart failure [17–19]. However, whether galectin-3 might be able to reflect cardiac function being assessed by MAPSE in patients undergoing cMRI has never been investigated.

Therefore, this study aims to investigate whether the biomarker of fibrosis galectin-3 might be able to reflect cardiac function being assessed by MAPSE in patients undergoing cMRI.

## 2. Methods

### 2.1. Study Population

The present study was conducted as a monocentric prospective study at the University Medical Center Mannheim (UMM), Germany. The study was carried out according to the principles of the declaration of Helsinki and was approved by the local ethics committee. Written informed consent was obtained from all participating patients or their legal representatives.

Patients undergoing cMRI during routine clinical care were included consecutively to this study from February 2015 until June 2015 within an all-comers design. In order to perform valuable cMRI examination all patients had to be in a stable clinical condition without acute clinical symptoms, such as acute dyspnea or extensive peripheral edema. The indications for cMRI were not restricted to any specific cardiac disease. Exclusion criteria for cMRI accorded to commonly known exclusion criteria, such as claustrophobia and metal implants [1]. Specifically for the present study, patients with a reduced RVF below 50% were excluded. All patients included were followed up to 6 and 12 months by standardized telephone visits.

All available clinical data of the study patients were documented, such as detailed findings of patients' prior medical history, laboratory findings, and medical therapies. Blood samples for biomarker measurements were collected once within 24 hours following the cMRI examination.

### 2.2. Measurements of Biomarkers

All samples were obtained by venipuncture into serum monovette® and centrifuged at 2500 ×g at 20°C for 10 minutes. The aliquoted samples were stored at −80°C until analysis. After thawing the samples were mixed gently by inverting and centrifuged with 2500 ×g for 10 minutes.

Galectin-3 was measured with the Galectin-3 assay on an Architect i1000 analyzer (Abbott, Wiesbaden, Germany). The limit of blank for this assay was 0.8 ng/mL as described in the instructions for use [20]. NT-proBNP was measured with the proBNP II STAT assay on a cobas e 602 analyzer (Roche Diagnostics, Mannheim, Germany). The limit of detection (LoD) for this assay was 5 pg/mL [21]. Serum creatinine was measured with the Creatinine Jaffe Gen. 2 assay on a cobas c 702 analyzer (Roche Diagnostics, Mannheim, Germany).

### 2.3. cMRI Acquisition

All studies were performed using a 1.5-Tesla whole body imaging system (Magnetom Avanto and Sonata, Siemens Medical Systems, Healthcare Sector, Erlangen, Germany) using a four-element (Sonata) or six-element (Avanto) phased-array body coil.

Cine images were acquired using a retrospective electrocardiographic-gated, balanced segmented steady state free precession (trueFISP) sequence in three long-axis views (2-, 3-, and 4-chamber views) and in multiple short-axis views, covering the entire left ventricle from base to apex.

### 2.4. cMRI Analysis

The cMRI image analysis was performed using the commercially available computer software program cvi^42^® (Circle Cardiovascular Imaging Inc., Calgary, Canada). MAPSE measurements were assessed on four-chamber view cine images. The distance between the basal septal mitral annulus (septal MAPSE), the basal lateral mitral annulus (lateral MAPSE), and a reference point outside the left ventricular apex was measured in end-diastole and end-systole. The distance travelled by the septal and lateral annulus from end-diastole to end-systole was calculated as septal and lateral MAPSE by subtracting the left ventricular end-systolic length (LVESL) from the left ventricular end-diastolic length (LVEDL) as being described previously [5]. Average MAPSE was calculated as the average of septal and lateral MAPSEs. Three subgroups were built according to MAPSE (MAPSE I: ≥11 mm, MAPSE II: ≥8 mm–<11 mm, and MAPSE III: <8 mm).

### 2.5. Statistical Analysis

For data with normal distribution, the Student* t*-test was applied. Otherwise, Kruskal-Wallis-Test was used as nonparametric test. Deviations from a Gaussian distribution were tested by the Kolmogorov–Smirnov test. Spearman's rank correlation for nonparametric data was used to test the association of galectin-3 blood levels with medical parameters and cardiac indices on cMRI. Data are presented as mean with confidence interval (CI) or median with interquartile ranges (IQR) (25th to 75th percentiles), depending on the distribution of the data. The *p* values <0.05 were considered statistically significant. To evaluate whether galectin-3 may reflect LVF being assessed by MAPSE better than NT-proBNP, the likelihoods for MAPSE of each marker were plotted and compared by the method of Hanley and McNeil [22]. To evaluate the potential confounding factors multivariable linear or logistic regression analyses with backward elimination were performed with adjustment of several clinical parameters or biomarkers depending on the outcome variable (being binary or numeric). Statistical analyses were performed in all patients and in three subgroups according to MAPSE (MAPSE I: ≥11 mm; MAPSE II: ≥8 mm–<11 mm; and MAPSE III: <8 mm). Cutoffs of biomarkers were set at the group specific medians of each biomarker for the groups of reduced MAPSE. The calculations were performed with GraphPad Prism (GraphPad Software) and SPSS software (SPSS Software GmbH).

## 3. Results

### 3.1. Study Population

A total of 84 chronic heart failure patients were enrolled in the present study (Table 1). Median age of the patients was 55 years (range 18–85 years). Most patients were of male gender (69%). 38% of patients suffered from compensated CHF (according to LVEF < 55%) with only mild to moderate symptoms according to NYHA classes I and II (*n* = 24, 92% of CHF patients). 37% of patients revealed valvular heart diseases, such as mitral valve (18%), tricuspid (12%), and aortic (6%) valve regurgitation, being mostly of mild and less often of moderate grade. Severe mitral valve regurgitation and aortic valve stenosis were present only in one patient each. 31% of patients presented with coronary artery disease, of which 23% had aortic coronary bypass grafts (ACVB) operation (*n* = 6). Seven patients (8%) suffered from chronic kidney disease.

According to the extent of MAPSE, three subgroups were defined as follows: MAPSE I ≥ 11 mm (*n* = 35, 42%), MAPSE II 8–11 mm (*n* = 31, 37%), and MAPSE III < 8 mm (*n* = 18, 21%).

### 3.2. Distribution of Cardiac MRI Indices according to MAPSE Subgroups

Median LVEF was 59% (IQR 51–64%) in the total cohort (Table 2). LVEF decreased significantly according to impaired subgroups of MAPSE (*p* = 0.007). Despite the exclusion of patients with RV dysfunction (RVF < 50%), tricuspid annular plane systolic excursion (TAPSE) decreased significantly alongside with impaired MAPSE (*p* = 0.0001). No significant differences were observed for remodeling index, RV ejection fraction (RVEF), septal wall thickness (SWT), and posterior wall thickness (PWT) alongside with reduced MAPSE (*p* > 0.05) (Table 2).

### 3.3. Galectin-3 and NT-proBNP in MAPSE Subgroups

**Figure 1:**
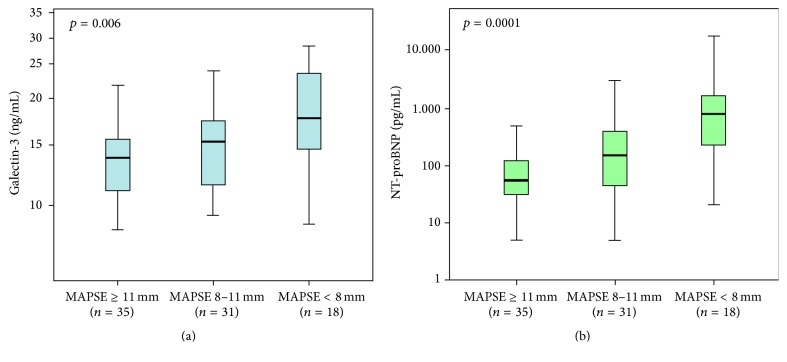
Distribution of galectin-3 (a) and NT-proBNP (b) serum levels according to subgroups of reduced MAPSE. Data are presented as medians with 25th and 75th percentiles (boxes) and 5th and 95th percentiles (whiskers).

Figures 1(a) and 1(b) demonstrate significantly increasing galectin-3 and NT-proBNP levels according to subgroups of decreased MAPSE (*p* = 0.006, *p* = 0.0001). Galectin-3 levels were as follows: MAPSE I (median 13.50 ng/mL, IQR 10.60–15.30 ng/mL), MAPSE II (median 15.00 ng/mL, IQR 11.20–17.20 mg/mL), and MAPSE III (median 17.50 ng/mL, IQR 13.93–23.55 ng/mL). NT-proBNP levels were as follows: MAPSE I (median 55.68 pg/mL, IQR 31.29–134.90 pg/mL), MAPSE II (median 151.80 pg/mL, IQR 38.34–406.80 pg/mL), and MAPSE III (median 808.05 pg/mL, IQR 229.43–2285.75 pg/mL).

After exclusion of patients with a reduced LVEF < 55% (31%), galectin-3 increased alongside with impaired subgroups of MAPSE (statistical trend, *p* = 0.1), whereas a univariable correlation between galectin-3 and MAPSE was no longer significant.

### 3.4. Correlations of Galectin-3 with cMRI Parameters

Galectin-3 levels correlated significantly with patients' age in MAPSE I (*r* = 0.43, *p* = 0.01) and MAPSE II (*r* = 0.52, *p* = 0.003), but not with serum creatinine (Table 3). Galectin-3 was only correlated with LVEF in all patients (*r* = −0.23; *p* = 0.02), whereas no significant correlations were found in the three subgroups of reduced MAPSE (*p* > 0.05). Despite exclusion of RV dysfunction, RV volumes being referred to body surface area (BSA) were in part associated with galectin-3. No other significant correlations were found between galectin-3 and cMRI parameters, such as RVEF and remodeling index. Noteworthy, galectin-3 correlated significantly with NT-proBNP levels in all patients (*r* = 0.42, *p* = 0.0001) and in those with most reduced MAPSE of <8 mm (*r* = 0.48, *p* = 0.04).

### 3.5. Galectin-3 Discriminates Reduced MAPSE

**Figure 2:**
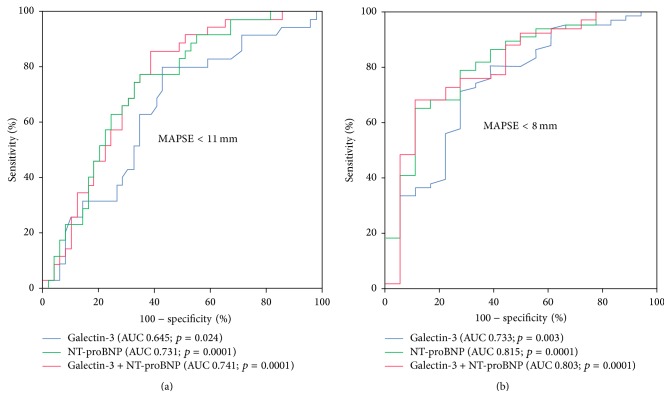
Receiver-operating characteristic (ROC) curves revealing valuable discrimination of patients with reduced MAPSE of < 11 mm (a) and < 8 mm (b) by serum levels of galectin-3.

As being analyzed by receiver-operating characteristic (ROC) curve analyses, galectin-3 levels discriminated patients with reduced MAPSE < 11 mm from all others (area under the curve (AUC) = 0.645, 95% CI 0.52–0.76, *p* = 0.024) (Figure 2(a)). In contrast, NT-proBNP revealed a numerically greater AUC compared to galectin-3 (AUC = 0.731, 95% CI 0.62–0.83, *p* = 0.0001) (Figure 2(a)). Combining galectin-3 with NT-proBNP revealed best discrimination of patients with MAPSE lower than 11 mm (combined AUC = 0.741; *p* = 0.0001). Additionally, both galectin-3 levels (AUC = 0.733, 95% CI 0.59–0.87, *p* = 0.003) and NT-proBNP (AUC = 0.815, 95% CI 0.72–0.92, *p* = 0.0001) discriminated patients with reduced MAPSE < 8 mm, whereas combining both biomarkers did not improve discrimination of this subgroup (Figure 2(b)).

### 3.6. Galectin-3 Reveals Independent Association with Reduced MAPSE

Galectin-3 levels were adjusted within multivariable logistic regression models for age, gender, creatinine, and NT-proBNP. In these multivariable logistic regression models, galectin-3 levels ≥ 16.2 ng/mL, corresponding to the median of patients with MAPSE < 11 mm, were 3-4 times more likely to suffer from MAPSE < 11 mm (adjusted odds ratio (OR) = 3.53, 95% CI 1.24–10.05, *p* = 0.018) (Table 4(a)). Patients with galectin-3 levels ≥ 17.5 ng/mL were 3 times more likely to be associated with reduced MAPSE < 8 mm (OR = 3.18, 95% CI 0.93–10.82, *p* = 0.06) (Table 4(b)). Accordingly, patients with increased NT-proBNP were up to 4–8 times more likely to suffer from reduced MAPSE (MAPSE < 11 mm: OR = 4.34, 95% CI 1.48–12.75, *p* = 0.007; MAPSE < 8 mm: OR = 8.50, 95% CI 2.34–30.86, *p* = 0.001) (Tables 4(a) and 4(b)).

## 4. Discussion

This study demonstrates that the biomarker of fibrosis galectin-3 is able to reflect MAPSE being assessed by cMRI. Galectin-3 was inversely correlated with MAPSE. Highest galectin-3 levels were associated with most impaired MAPSE < 8 mm. Even after adjustment with clinical confounding factors as well as NT-proBNP, increased galectin-3 levels were still significantly associated with impaired MAPSE. Combining galectin-3 with NT-proBNP improved the discriminative capacity to detect impaired MAPSE < 11 mm. To the best of our knowledge, the present study is the first investigating galectin-3 levels to assess LVF corresponding to MAPSE in patients undergoing cMRI.

Numerous experimental studies have demonstrated recently that galectin-3 might contribute to the pathophysiology of an adverse structural remodeling within the development of heart failure [23]. Fibroblasts and myofibroblasts are considered as key cells being responsible for the initiation and progression of tissue scarring [24]. Furthermore, activation and infiltration of macrophages within the myocardium were also shown to be associated with an adverse cardiac remodeling [25, 26]. Galectin-3 is upregulated rapidly in hypertrophied hearts and released by paracrine effects from the epithelium and inflammatory cells, especially activated cardiac macrophages [27]. Increased galectin-3 was shown to stimulate macrophages migration itself through the release of transforming growth factor- (TGF-) beta and interleukin-1 or -2. Taken together, galectin-3 induces the proliferation of myofibroblasts and collagen disposition and thereby influences ventricular dysfunction [12, 28, 29]. Sharma et al. evaluated in an animal model with transgenic Ren-2 rats the gene expression of galectin-3 using a complementary DNA array with whole RNA from myocardial biopsies during the progression of heart failure related to renin-dependent hypertension and found an increased myocardial galectin-3 expression in those rats developing heart failure compared to those without [12]. de Boer et al. indicated that an early increase in galectin-3 expression identified failure-prone hypertrophied hearts [30]. Liu et al. demonstrated that pericardial infusion of galectin-3 enhanced macrophage as well as mast cell infiltration and cardiac interstitial and perivascular fibrosis and caused cardiac hypertrophy. TGF-beta expression and Smad3 phosphorylation were also induced by galectin-3. Additionally, galectin-3 decreased also the ratio of early LV filling to atrial contraction phase as well as LVEF [31]. In contrast, genetic and pharmacological inhibition of galectin-3 was shown to prevent adverse cardiac remodeling [32].

MAPSE serves as a central parameter of cardiac function being measured by cMRI. MAPSE represents a direct measure of the ratio of longitudinal left ventricular wall contractility during systole and diastole, whereas LVEF represents the indirect ratio of LV volumes being assessed by circumferential planimetrics of the LV cavity. Therefore, MAPSE might reveal more subtle and direct contractility dysfunctions compared to LVEF [33]. It was shown that patients with a preserved ejection fraction (those with increasing age, hypertension, myocardial hypertrophy, or diastolic dysfunction) reveal an impaired long-axis contractile function earlier, even when radial function still remains preserved [34]. However, significant positive correlations were still found between MAPSE and LVEF irrespective of imaging technique, such as M-mode, Simpson's rule, visual estimation, on both transthoracic and three-dimensional echocardiography, or cMRI [35–37]. Furthermore, patients with reduced MAPSE of less than 5 mm revealed a higher long-term mortality rate than patients with MAPSE of more than 9 mm, even after adjustment in multivariable Cox proportional hazard analyses [38]. Interestingly, Elnoamany and Abdelhameed showed that MAPSE correlated inversely with serum levels of NT-proBNP [10].

The present study combines the assessment of MAPSE by modern cMRI with a combination of two blood biomarkers of different pathophysiological backgrounds, that is, the natriuretic peptide NT-proBNP and the biomarker of fibrosis galectin-3. This diagnostic combination might reflect LV dysfunction already in patients at very early and compensated stages of CHF with only mild to moderate symptoms being present in this study cohort. The present clinical finding might be supported by the mentioned experimental data, indicating that galectin-3 is already increased at early heart failure stages with beginning cardiac hypertrophy [12, 30]. Cardiac remodeling is nowadays considered as a key determinant for the development of heart failure, and one of the main therapeutic goals is to retard or even reverse adverse structural remodeling in order to prevent the development of severe stages of heart failure [39]. Combining modern cardiac imaging with a combination of reliable cardiac biomarkers might bear the potential to improve the early diagnosis and risk stratification of patients with early stages of LV dysfunction in the upcoming future.

## Figures and Tables

**Table 1:** Baseline characteristics of study patients (*n* = 84).

Characteristic	Values
Age, median (range; 95% CI^a^)	55.2 (18–85; 51.6–58.8)
Gender, *n* (%)	
Male	58 (69)
Female	26 (31)
Cardiovascular risk factors, *n* (%)	
Arterial hypertension	37 (44)
Hypercholesterinemia	21 (25)
Cardiac family history	15 (18)
Smoking status	32 (38)
Diabetes mellitus	11 (13)
Obesity	12 (14)
Laboratory parameters, median (IQR^b^)	
Creatinine (mg/dL)	0.89 (0.78–1.04)
GFR (mL/min)	89.00 (75.00–101.00)
Medical history, *n* (%)	
Chronic heart failure	32 (38)
NYHA I	10 (12)
NYHA II	14 (17)
NYHA III	7 (8)
NYHA IV	1 (1)
Atrial fibrillation	13 (15)
Paroxysmal	8 (10)
Persistent	3 (4)
Permanent	2 (2)
Coronary artery disease	26 (31)
1 vessel disease	10 (12)
2 vessel diseases	3 (4)
3 vessel diseases	13 (15)
Myocardial infarction	17 (20)
Valvular heart disease	31 (37)
Chronic kidney disease	7 (8)
COPD	7 (8)
Asthma	6 (7)
Pneumonia	2 (2)
Pulmonary hypertension	1 (1)
Cancer	7 (8)

^a^Confidence interval.

^b^Interquartile range.

**Table 2:** Distribution of cardiac MRI indices according to MAPSE subgroups.

	MAPSE I ≥ 11 mm (*n* = 35)	MAPSE II 8–11 mm (*n* = 31)	MAPSE III < 8 mm (*n* = 18)	*p* value
LVEF	61.00 (56.00–66.00)	57.00 (45.00–61.00)	57.00 (36.00–60.50)	**0.007**
LVEDV/BSA^a^	91.66 (80.08–103.18)	82.93 (66.46–92.14)	82.42 (68.03–105.27)	0.05
LVESV/BSA^a^	36.05 (27.71–42.14)	36.00 (25.30–50.02)	34.78 (24.81–65.07)	0.76
LVSV/BSA^a^	55.00 (48.80–61.88)	44.04 (38.73–50.55)	38.68 (33.52–49.86)	**0.0001**
RVEF	61.89 (55.42–64.67)	61.46 (56.07–66.92)	64.12 (57.09–66.89)	0.51
RVEDV/BSA^a^	86.39 (79.25–94.19)	66.15 (59.85–76.50)	66.04 (44.11–75.31)	**0.0001**
RVESV/BSA^a^	33.77 (28.66–39.18)	25.81 (20.20–30.46)	23.93 (15.22–33.04)	**0.0001**
RVSV/BSA^a^	52.60 (45.60–59.21)	41.57 (35.47–46.49)	39.72 (28.89–49.23)	**0.0001**
TAPSE	2.16 (1.89–2.41)	1.68 (1.45–2.05)	1.41 (0.77–1.99)	**0.0001**
Remodeling index	0.78 (0.65–0.90)	0.79 (0.70–0.89)	0.97 (0.76–1.18)	0.063
PWT	7.00 (6.00–7.00)	6.00 (6.00–7.00)	8.00 (6.00–9.00)	0.05
SWT	10.00 (8.00–12.00)	9.00 (8.00–11.00)	10 (8.00–14.25)	0.59

LVEF, left ventricular ejection fraction; LVEDV, LV end-diastolic volume; LVESV, LV end-systolic volume; LVSV, LV stroke volume; RVEF, right ventricular ejection fraction; RVEDV, RV end-diastolic volume; RVESV, RV end-systolic volume; RVSV, RV stroke volume; TAPSE, tricuspid annular posterior systolic excursion; PWT, posterior wall thickness; SWT, septal wall thickness.

^a^Body surface area.

Data presented as median with interquartile ranges (IQR).

Bold values indicate statistically significant p values (*p* < 0.05).

**Table 3:** Univariable correlations between galectin-3 and baseline characteristics, biomarkers, and cardiac MRI parameters according to MAPSE subgroups.

	All patients (*n* = 84)		MAPSE I ≥ 11 mm (*n* = 35)		MAPSE II 8–11 mm (*n* = 31)		MAPSE III < 8 mm (*n* = 18)	
	*r*	*p* value	*r*	*p* value	*r*	*p* value	*r*	*p* value
Age	0.41	**0.0001**	0.43	**0.01**	0.52	**0.003**	−0.05	0.86
Creatinine	0.18	0.09	0.07	0.68	0.06	0.75	0.31	0.21
NT-proBNP	0.42	**0.0001**	0.19	0.28	0.27	0.14	0.48	**0.04**
LVEF	−0.23	**0.02**	−0.25	0.15	0.11	0.55	−0.36	0.15
LVEDV/BSA^a^	−0.03	0.72	−0.24	0.16	−0.19	0.28	0.55	**0.01**
LVESV/BSA^a^	0.09	0.38	0.02	0.90	−0.19	0.29	0.42	0.07
LVSV/BSA^a^	−0.27	**0.01**	−0.40	**0.01**	−0.21	0.24	0.28	0.25
RVEF	0.25	**0.01**	−0.04	0.81	0.54	**0.002**	−0.02	0.93
RVEDV/BSA^a^	−0.37	**0.0001**	−0.52	**0.001**	−0.49	**0.005**	0.36	0.14
RVESV/BSA^a^	−0.39	**0.0001**	−0.28	0.108	−0.57	**0.001**	0.21	0.41
RVSV/BSA^a^	−0.24	**0.02**	−0.37	**0.028**	−0.23	0.22	0.40	0.10
TAPSE	−0.16	0.13	−0.03	0.86	0.10	0.58	−0.05	0.84
Remodeling index	−0.05	0.63	0.001	0.997	−0.14	0.47	−0.33	0.18
PWT	0.08	0.42	0.33	**0.04**	−0.08	0.64	−0.09	0.70
SWT	0.14	0.19	0.15	0.38	0.28	0.12	−0.12	0.63

LVEF, left ventricular ejection fraction; LVEDV, LV end-diastolic volume; LVESV, LV end-systolic volume; LVSV, LV stroke volume; RVEF, right ventricular ejection fraction; RVEDV, RV end-diastolic volume; RVESV, RV end-systolic volume; RVSV, RV stroke volume; TAPSE, tricuspid annular posterior systolic excursion; PWT, posterior wall thickness; SWT, septal wall thickness.

^a^Body surface area.

Bold values indicate statistically significant *p*  value (*p* < 0.05).

**Table 4:** (a) Multivariable logistic regression model for evaluating the ability of galectin-3 to identify patients with reduced MAPSE of <11 mm. (b) Multivariable logistic regression for evaluating the ability of galectin-3 to identify patients with reduced MAPSE of <8 mm.

	Adjusted odds ratio	95% CI^b^	Adjusted *p* value
(a)			

Galectin-3 (≥16.2 ng/mL)	3.53	1.24–10.05	**0.018**
NT-proBNP (≥285.2 pg/mL)	4.34	1.48–12.75	**0.007**
Gender^a^	1.61	0.54–4.77	0.39
Age	0.98	0.95–1.02	0.49
Creatinine	0.84	0.12–5.69	0.86

(b)			

Galectin-3 (≥17.5 ng/mL)	3.18	0.93–10.82	0.06
NT-proBNP (≥808.0 pg/mL)	8.50	2.34–30.86	**0.001**
Age	0.98	0.94–1.03	0.51
Gender^a^	1.28	0.36–4.50	0.70
Creatinine	1.19	0.33–4.25	0.77

Bold values indicate statistically significant *p* values (*p* < 0.05).

^a^An adjusted odds ratio of >1 indicates an association of male gender with reduced MAPSE.

^b^Confidence interval.
